# Retrospective cohort study of *Helicobacter pylori* infection and risk stratification using 6-year UBT data

**DOI:** 10.3389/fpubh.2025.1563841

**Published:** 2025-05-26

**Authors:** Yan Chen, Miaojuan Wang, Jianfeng Wang

**Affiliations:** ^1^Department of General Practice, The First Affiliated Hospital of Zhejiang Chinese Medical University, Zhejiang Provincial Hospital of Traditional Chinese Medicine, Hangzhou, China; ^2^Department of Respiratory Diseases, The First Affiliated Hospital of Zhejiang Chinese Medical University, Zhejiang Provincial Hospital of Traditional Chinese Medicine, Hangzhou, China; ^3^Institute of Respiratory Diseases of Traditional Chinese Medicine, Zhejiang Chinese Medical University, Hangzhou, China

**Keywords:** *Helicobacter pylori*, urea breath test, metabolic syndrome, machine learning, XGBoost, risk stratification, longitudinal study, chronic infection

## Abstract

**Background:**

*Helicobacter pylori* (*H. pylori*) infection is a major global health concern, linked to gastric cancer and metabolic disorders. Despite its widespread prevalence, accurate risk stratification remains challenging. This study aims to develop a machine learning (ML)-based risk prediction model using 6-year longitudinal Urea Breath Test (UBT) data to identify metabolic alterations associated with chronic *H. pylori* infection.

**Methods:**

A retrospective cohort study was conducted using health examination data from 3,409 individuals between 2016 and 2021. Participants were stratified into *H. pylori*-positive and negative groups based on longitudinal UBT results. Key metabolic markers, including HbA1c, LDL-C, BMI, and WBC, were analyzed. Three predictive models—logistic regression, random forest, and XGBoost—were compared to assess their predictive performance.

**Results:**

Among the cohort, 20.5% exhibited chronic *H. pylori* infection. Infected individuals had significantly higher HbA1c (+1.2%, *p* < 0.01), LDL-C (+15 mg/dL, *p* < 0.05), and WBC levels, alongside lower albumin (−0.8 g/dL, *p* < 0.01). The XGBoost model outperformed others (AUC = 0.6809, Accuracy = 81.13%) in predicting infection risk. A subgroup of 4.0% was identified as high-risk, highlighting the potential for early intervention.

**Conclusion:**

This study underscores the interplay between chronic *H. pylori* infection and metabolic dysfunction, offering new perspectives on risk prediction using machine learning. The XGBoost model demonstrated reliable performance in stratifying infection risk based on accessible clinical markers. Its integration into routine screening protocols could enhance early detection and personalized intervention strategies. Further studies should validate these findings across broader populations and incorporate additional risk factors.

## Introduction

Gastric cancer remains a major public health burden in China, where incidence and mortality rates are among the highest globally. In Xiamen alone, age-standardized rates reached 16.74 and 12.30 per 100,000 between 2011 and 2020 (1). Given that East Asia accounts for more than half of global gastric cancer cases, a focused investigation within the Chinese population holds significant value for targeted prevention strategies (2).

*Helicobacter pylori* (*H. pylori*), designated by the WHO as a Group 1 carcinogen, is a leading contributor to peptic ulcer disease and gastric malignancy (3). Its persistent colonization has also been linked to systemic conditions including anemia, cardiovascular disorders, and autoimmune diseases (4, 5). Eradication therapy, when administered early, effectively reduces gastric cancer risk, yet untreated infections may persist for decades (6).

Beyond gastrointestinal complications, emerging evidence suggests a role for *H. pylori* in metabolic syndrome (MetS), a cluster of conditions encompassing obesity, hyperglycemia, hypertension, and dyslipidemia (7). Cross-sectional and animal studies indicate that *H. pylori* infection disrupts glucose and lipid metabolism (8), potentially exacerbating metabolic disorders. Intervention studies further support that bacterial eradication can improve glycemic and lipid parameters (9).

Machine learning (ML), a branch of artificial intelligence, leverages algorithms to identify complex patterns in large datasets and generate predictive models without explicit programming (10). In biomedical research, techniques such as support vector machines, random forests, and deep neural networks have been successfully applied to diagnostic classification, risk stratification, and outcome prediction. Notably, convolutional neural network–based AI systems have demonstrated high sensitivity and specificity in analyzing endoscopic images to detect *H. pylori*–related mucosal changes, enabling real-time, noninvasive diagnosis and improved lesion characterization (8).

Among various machine learning algorithms, XGBoost has gained wide recognition for its superior accuracy, scalability, and ability to model complex nonlinear relationships. It employs gradient boosting framework and regularization techniques that reduce overfitting and enhance generalization, making it especially suitable for biomedical risk prediction involving heterogeneous clinical data. Its proven performance in infection risk modeling and early disease detection has established it as a preferred model in recent translational studies (11).

The present study aims to develop and validate an XGBoost-based model to predict chronic *H. pylori* infection and stratify gastric cancer risk using a 6-year longitudinal dataset from a Chinese cohort, integrating both infection status and metabolic profiles.

## Methods

### Study population

This retrospective cohort study was conducted at The First Affiliated Hospital of Zhejiang Chinese Medical University in Hangzhou, Zhejiang Province, China. We included individuals aged ≥18 years who underwent annual physical examinations and completed both ^13^C-UBT and ^14^C-UBT between January 2016 and December 2021. Patients with prior *H. pylori* eradication therapy before the initial UBT, malignancy, severe cardiovascular disease, gastrointestinal surgery, or incomplete UBT data were excluded. Follow-up UBTs were performed annually to assess chronic infection status.

The ^13^C-UBT was performed using the HCBT-01 device, and the ^14^C-UBT using the HUBT-01 device, both manufactured by Anhui Yanghe Medical Equipment Co., Ltd. under commission from Shenzhen Zhonghe Haidewei Biotechnology Co., Ltd. While both tests are based on the principle that *Helicobacter pylori* produces urease to hydrolyze urea into ammonia and carbon dioxide, ^13^C is a non-radioactive stable isotope, whereas ^14^C is a radioactive isotope. Patients chose between the two tests based on personal preference. All UBT results were recorded as quantitative values.

A positive UBT result was determined as follows: a ^14^C-UBT test exceeding 100 dpm or a ^13^C-UBT test surpassing 4DOB. Whenever the UBT value approached the defined threshold, a repeat test was administered to confirm the outcome. Subsequently, the study population was stratified into two distinct groups based on their UBT results. The negative group comprised participants who consistently tested negative, while the positive group included individuals who maintained consistently positive UBT results throughout a 6-year period, thus categorizing them as the *H. pylori* chronic infection group.

Each participant underwent comprehensive anthropometric and laboratory assessments encompassing 18 key indicators, including body mass index (BMI), systolic and diastolic blood pressure, serum total protein, serum globulin, serum albumin, alanine aminotransferase, aspartate aminotransferase, serum total cholesterol, triglycerides, HDL and LDL cholesterol levels, blood urea nitrogen, creatinine, uric acid, HbA1c, hemoglobin, and white blood cell count (WBC).

BMI was computed as the ratio of body weight in kilograms to the square of height in meters (kg/m^2^). Blood pressure measurements were taken three times with the subject in a seated position, and the average of the last two measurements was recorded. Blood analyses were conducted using a fully automated biochemical analyzer (Rochecobas 8,000), with all measurements performed by skilled medical professionals.

### Data preprocessing and model development

Continuous variables were standardized using Z-score standardization. The dataset was divided into a training set (70%) and a test set (30%). In the model development phase, we built three prediction models: a regular logistic regression model, a random forest model, and an XGBoost algorithm model. Model development followed a structured approach. Initially, significant variables were identified using backward stepwise selection through chi-square tests, applying an inclusion criterion of *p* < 0.05. Three predictive models were evaluated for clinical utility, with XGBoost demonstrating optimal performance. Key metabolic biomarkers (e.g., HbA1c, LDL-C) were prioritized using interpretability frameworks.

For the XGBoost model, we utilized the gradient boosting decision-tree framework implemented in the XGBoost library. Key hyperparameters were configured as follows: learning rate (eta) = 0.1, maximum tree depth = 6, subsample ratio = 0.8, and colsample bytree = 0.8. The model was trained for up to 100 boosting rounds with early stopping set to 10 rounds based on log-loss evaluation on a held-out validation set to prevent overfitting. We specified the objective function as binary:logistic and used log-loss as the primary evaluation metric.

### Assessment indicators and risk stratification

The predictive performance of the three models was compared using Receiver Operating Characteristic (ROC) curves and accuracy on the test dataset. Prediction of infection probability using XGBoost model. Individuals were classified as: Low risk: probability < 30%. Medium risk: 30% ≤ probability ≤ 70%. High risk: probability > 70%.

### Statistical analysis

Normally distributed continuous variables were presented as mean ± standard deviation (SD), and independent samples *t*-tests were employed for intergroup comparisons. Categorical variables were compared between groups using the chi-square test. To explore the association between variables and *H. pylori* infection, logistic regression analysis was carried out, yielding odds ratios (OR) and 95% confidence intervals (95% CI). All statistical analyses were performed using R version 4.2.1. Statistical significance was defined as a two-sided *p* < 0.05. Data visualization was performed using Python 3.13.1.

### Ethical approval

This study received approval from the Ethics Committee of the First Affiliated Hospital of Zhejiang University of Chinese Medicine (Approval number: 2023-K-254-01). Informed consent was waived since the data collected in this study did not contain confidential participant information. Our ethics committee has duly approved the consent waiver.

### Data preprocessing and model development

**Figure 1:**
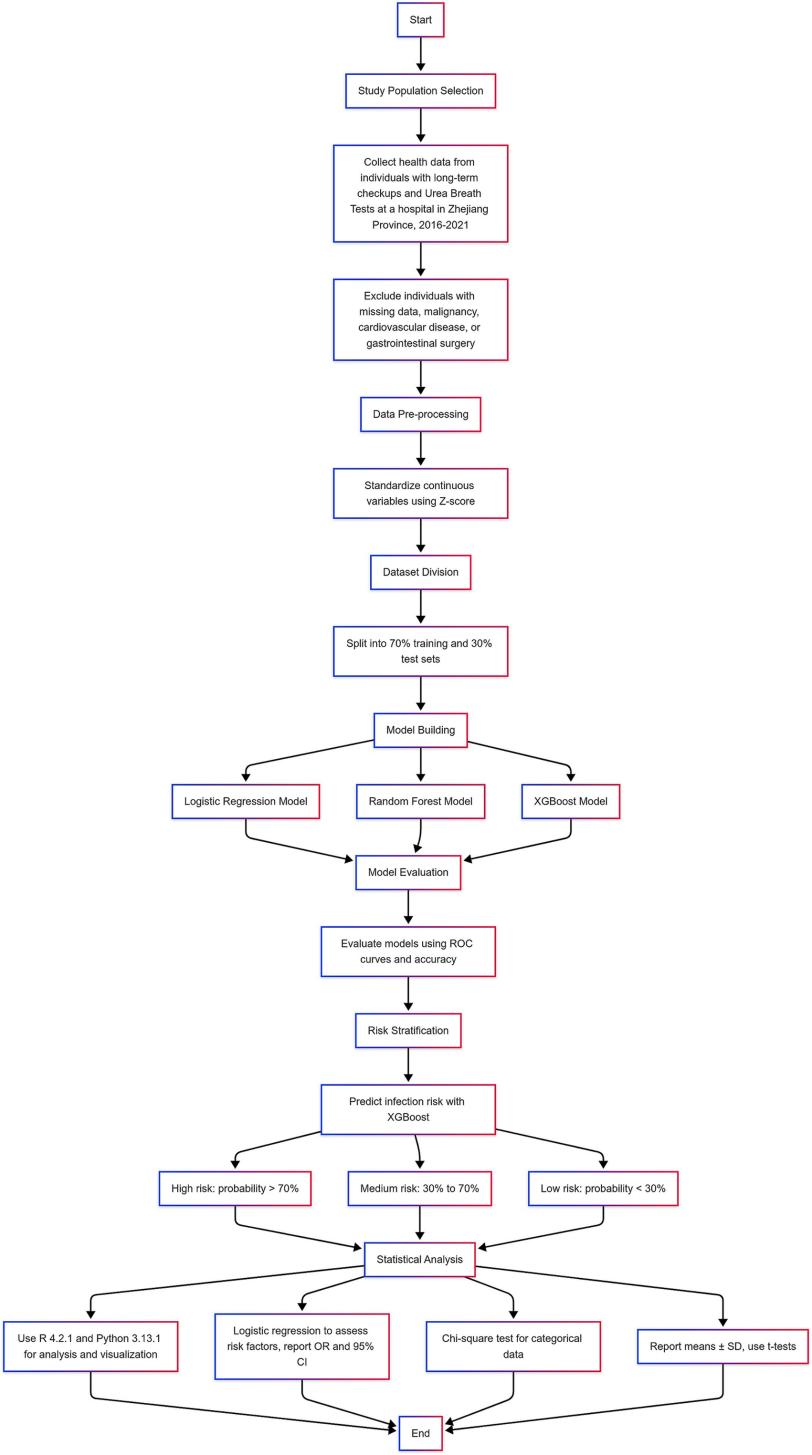
Flowchart of the study design and analysis process, including study population selection, exclusion criteria, data preprocessing, model construction and evaluation, risk stratification, and statistical analysis.

A visual summary of the study design, data processing steps, model development, evaluation, and risk stratification is presented in the following flowchart (Figure 1). This diagram helps clarify the overall analytical pipeline from patient selection to final statistical analysis.

## Results

### Study population characteristics

A total of 3,409 participants were included, comprising 1,979 males (58.1%) and 1,430 females (41.9%) (Table 1).

**Table 1:** The laboratory parameters results and statistical results.

Parameter	Negative group (Mean ± SD)	Positive group (Mean ± SD)	*P*-value
Gender (n, %)			
Male	1,563 (45.8%)	416 (12.2%)	0.376
Female	1,148 (33.7%)	282 (8.3%)	
Age (years)	44.8 ± 15.94	46.62 ± 15.59	0.007^*^
BMI (kg/m²)	23.87 ± 3.38	24.23 ± 3.45	0.013^*^
SBP (mm Hg)	122.34 ± 17.99	122.97 ± 18.03	0.409
DBP (mm Hg)	72.45 ± 10.59	72.61 ± 10.70	0.711
Total protein (g/L)	73.17 ± 3.89	72.70 ± 3.88	0.005^*^
Globulin (g/L)	24.90 ± 3.48	24.91 ± 3.52	0.946
Albumin (g/L)	48.28 ± 4.48	47.85 ± 4.72	0.0249^*^
ALT (U/L)	22.97 ± 26.09	23.08 ± 18.91	0.911
AST (U/L)	20.16 ± 14.58	19.99 ± 8.30	0.774
Total cholesterol (mmol/L)	4.58 ± 0.87	4.71 ± 0.92	0.001^*^
Triglycerides (mmol/L)	1.55 ± 1.27	1.63 ± 1.28	0.123
HDL-C (mmol/L)	1.32 ± 0.32	1.32 ± 0.32	0.916
LDL-C (mmol/L)	2.47 ± 0.68	2.55 ± 0.72	0.007^*^
BUN (mmol/L)	4.59 ± 1.17	4.65 ± 1.18	0.221
Creatinine (µmol/L)	71.41 ± 16.27	72.22 ± 17.95	0.250
Uric acid (µmol/L)	352.71 ± 89.01	352.92 ± 91.87	0.957
HbA1c (%)	5.74 ± 0.56	5.93 ± 0.89	<0.001^*^
Hb (g/L)	143.99 ± 15.56	144.51 ± 15.61	0.429
WBC (×10⁹/L)	6.12 ± 1.65	6.42 ± 1.59	<0.001^*^

BMI, body mass index; SBP, systolic blood pressure; DBP, diastolic blood pressure; ALT, alanine aminotransferase; AST, aspartate aminotransferase; HDL-C, high-density lipoprotein; LDL-C, low-density lipoprotein; BUN, urea nitrogen; HbA1c, glycosylated hemoglobin; Hb, hemoglobin; WBC, white blood cell. *Indicates *p* < 0.05.

Based on six consecutive years of UBT results, 2,711 individuals (79.5%) were classified as negative and 698 (20.5%) as positive, indicating chronic *H. pylori* infection.

Among the negative group, 1,563 were male (45.8% of total) and 1,148 female (33.7%); in the positive group, 416 were male (12.2%) and 282 female (8.2%). There was no significant difference in gender distribution between groups (*p* > 0.05).

The mean age was 44.8 ± 15.94 years in the negative group and 46.62 ± 15.59 years in the positive group, with the latter being slightly older (*p* < 0.05).

### Metabolic and laboratory profiles

As shown in Table 1, the chronic infection group exhibited significant alterations in metabolic markers compared to the negative group (*p* < 0.05):

Elevated HbA1c: +1.2% (*p* < 0.01).

Higher LDL-C: +15 mg/dL (p < 0.05).

Lower serum albumin: −0.8 g/dL (p < 0.01).

These findings align with inflammation-mediated metabolic disruption in chronic *H. pylori* infection.

### Correlation analysis among variables

Independent Pearson correlation analyses revealed moderate associations (|r| > 0.3) between:

Age and systolic blood pressure (SBP).

SBP and diastolic blood pressure (DBP).

Serum total protein and albumin (and albumin/globulin ratio).

Alanine aminotransferase (ALT) and aspartate aminotransferase (AST).

Total cholesterol and triglycerides.

Total cholesterol and LDL-C.

**Figure 2:**
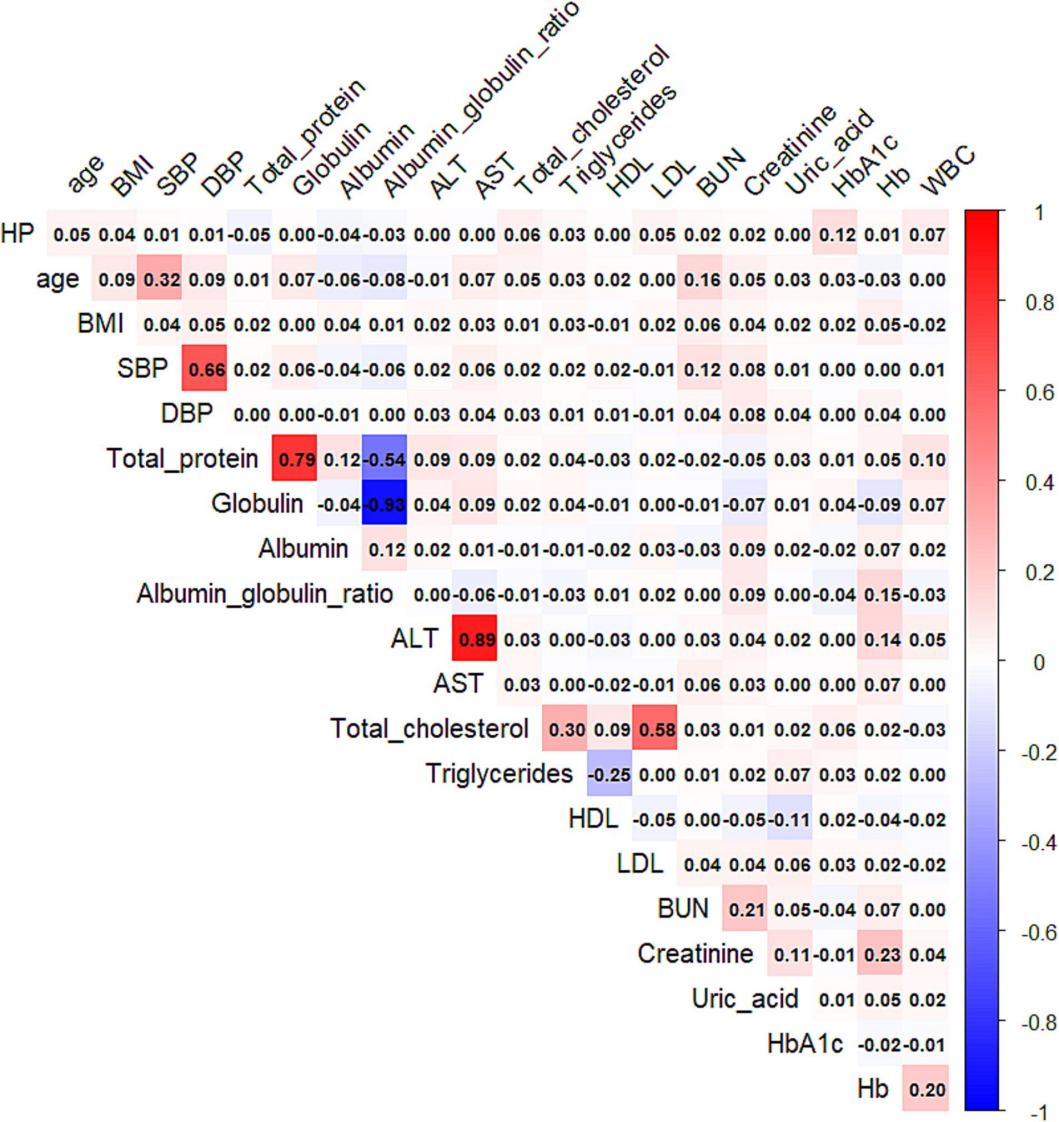
Heatmap of the Pearson correlation coefficients among the selected clinical and metabolic variables. The color gradient represents the strength and direction of the correlation, ranging from negative (blue) to positive (red). Only numeric variables were included in the analysis.

Most other variable pairs showed weaker correlations (|r| < 0.3), informing feature selection for model development (Figure 2).

### Model development and performance evaluation

Three predictive models were compared on the test dataset:

Logistic Regression: AUC = 0.6357, Accuracy = 79.86%.

Random Forest: AUC = 0.6790, Accuracy = 80.94%.

XGBoost: AUC = 0.6809, Accuracy = 81.13%.

**Figure 3:**
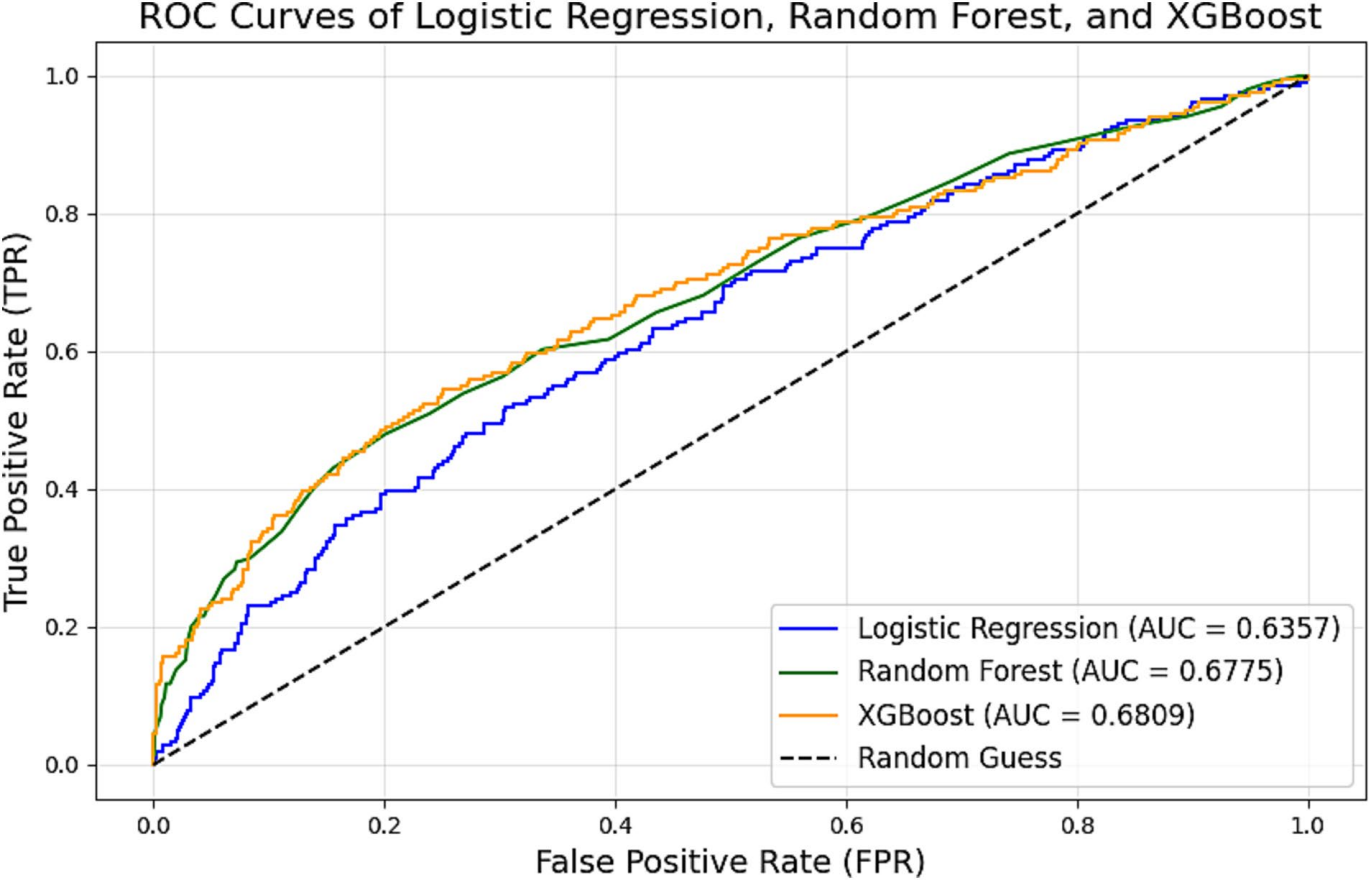
The area under the receiver operating characteristic curve (AUC) values for the logistic regression, random forest, and XGBoost models are 0.6357, 0.6790, and 0.6809, respectively.

**Figure 4:**
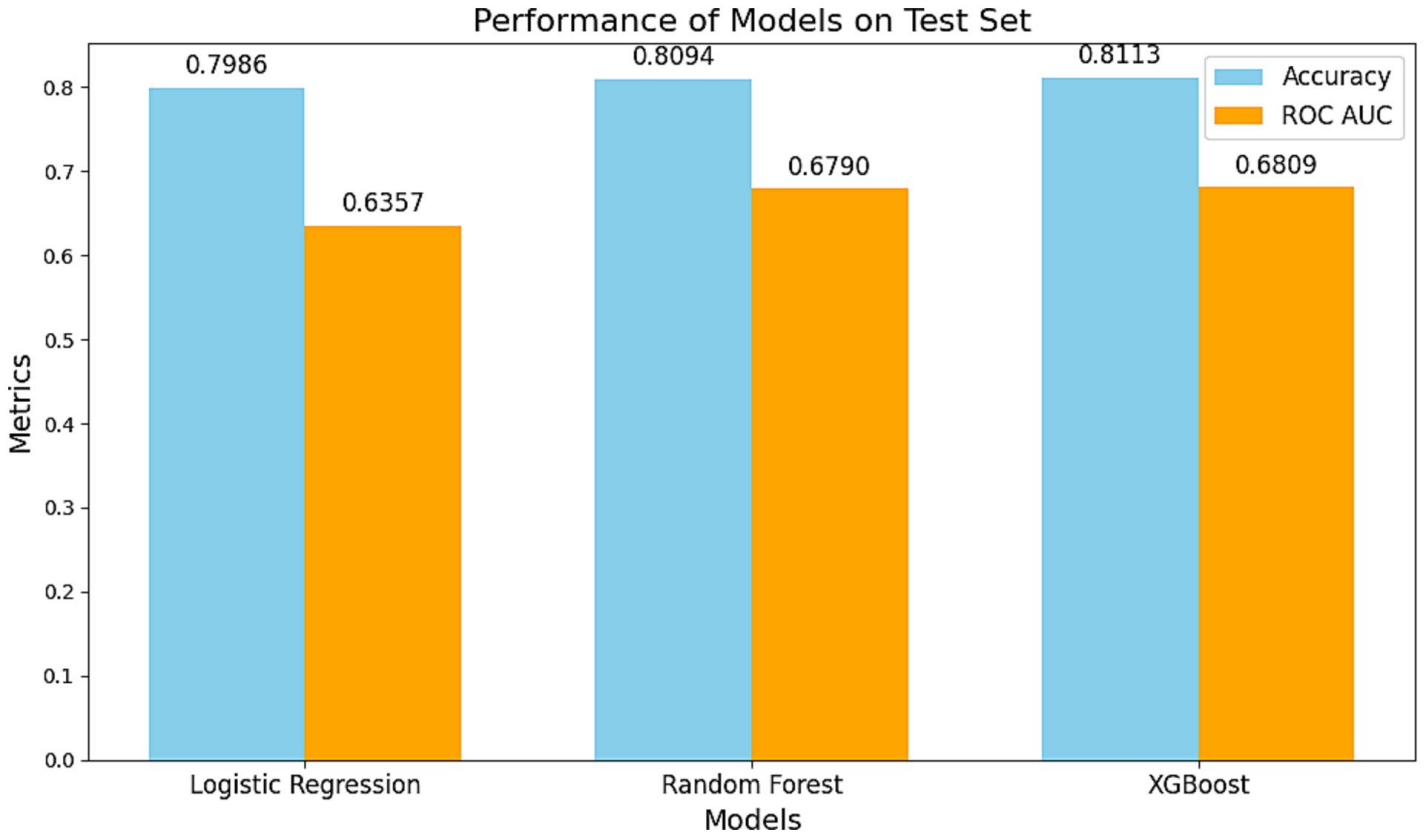
Comparison of the performance of different models on the test set. The blue bars represent model accuracy, while the orange bars indicate the AUC of the ROC curve. The XGBoost model demonstrated the best performance with an accuracy of 0.8113 and an AUC of 0.6809, highlighting its superiority in handling non-linear associations and complex interactions. The Random Forest model ranked second, while the Logistic Regression model exhibited stable performance in capturing linear relationships (Accuracy: 0.7986, AUC: 0.6357).

**Figure 5:**
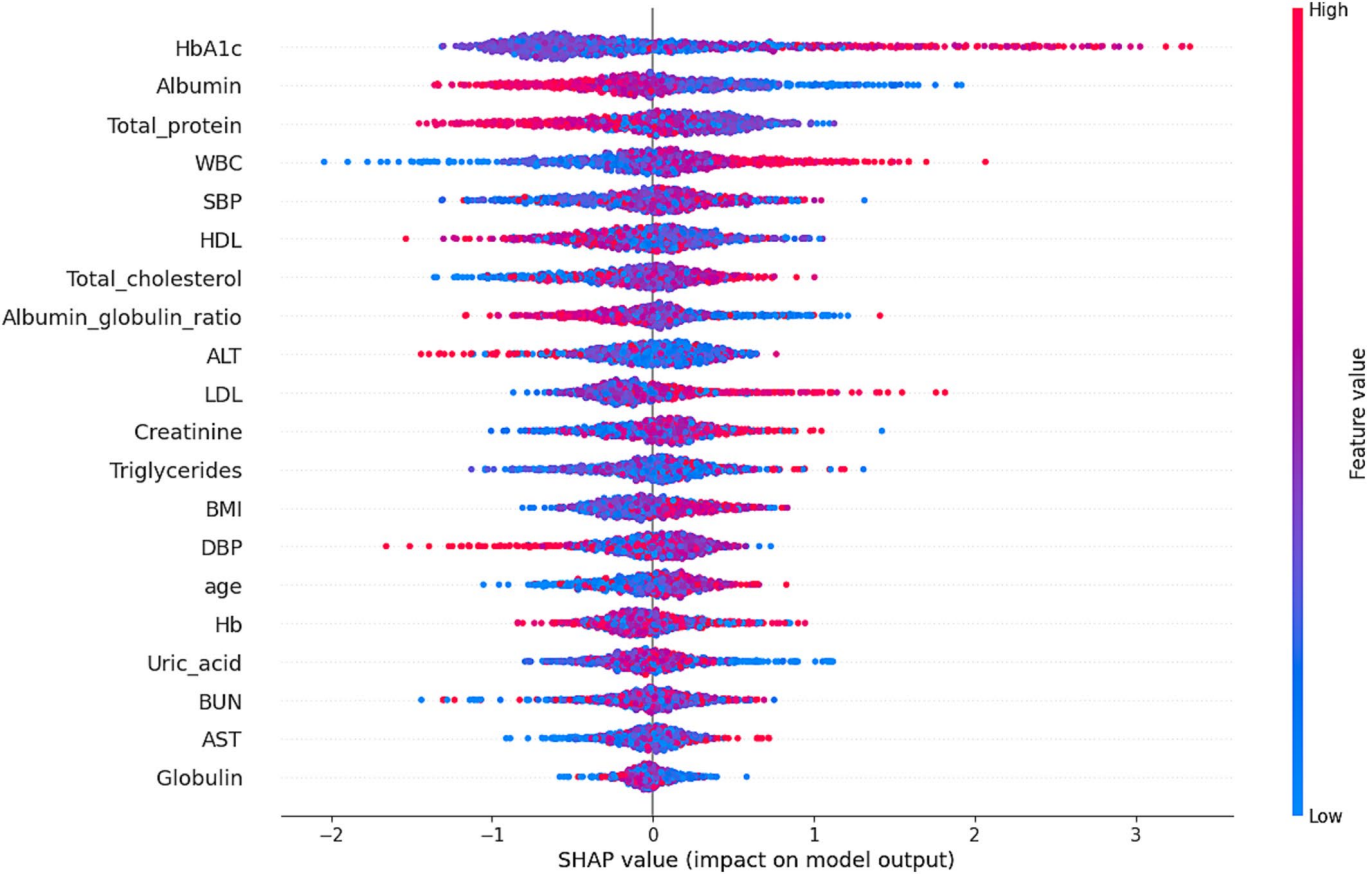
Shapley additive explanations (SHAP) values illustrate the contribution of each feature to the risk of *H. pylori* infection as predicted by the XGBoost model. The x-axis represents the SHAP value, indicating the impact of each feature on the model’s predictions: positive SHAP values increase infection risk, while negative values reduce it. The color of the points reflects feature values, with red indicating high values and blue indicating low values. For instance, HbA1c and Albumin exhibited significant non-linear effects on infection risk, with their impact varying across different value ranges. The model achieved an accuracy of 81.13% and an AUC of 68.09%, demonstrating its effectiveness in risk prediction.

The XGBoost model outperformed the others in both accuracy and AUC, demonstrating its robustness in capturing complex, non-linear relationships (Figures 3–5).

### Infection probability distribution and risk stratification

Using the XGBoost–predicted infection probabilities, participants were stratified into three risk categories:

Low risk (< 30%): 884 individuals (86.4%).

Medium risk (30–70%): 98 individuals (9.6%).

High risk (> 70%): 41 individuals (4.0%).

**Figure 6:**
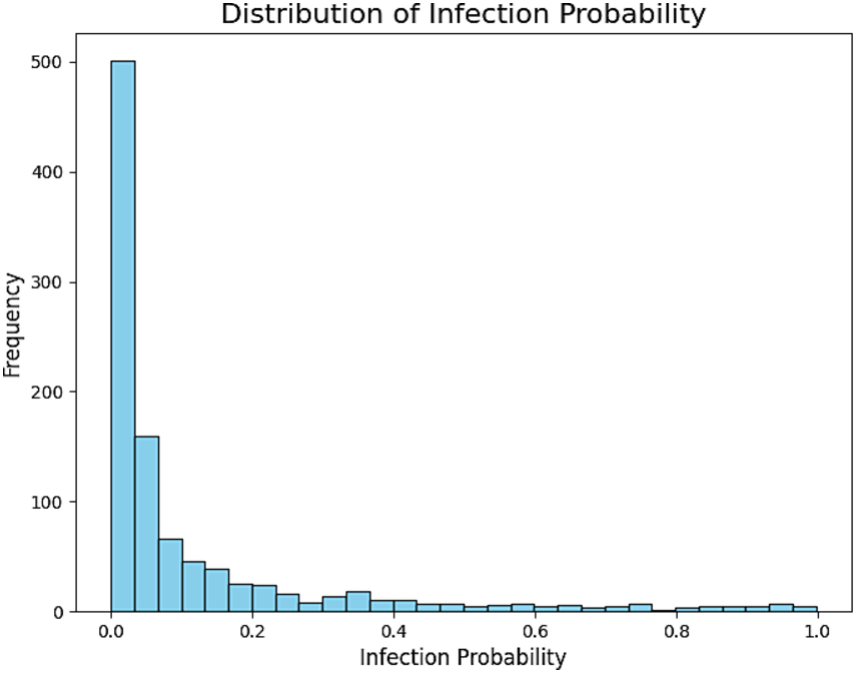
This histogram depicts the distribution of predicted probabilities for *H. pylori* infection in the test set. The x-axis shows the predicted probability range (from 0 to 1), while the y-axis represents the number of individuals in each range. Most individuals had predicted probabilities concentrated in the lower range (<0.3), indicating that the majority of predictions fall into the low-risk category. Medium-risk (0.3–0.7) and high-risk (>0.7) individuals were relatively fewer, aligning with the real-world distribution where healthy individuals constitute the majority.

**Figure 7:**
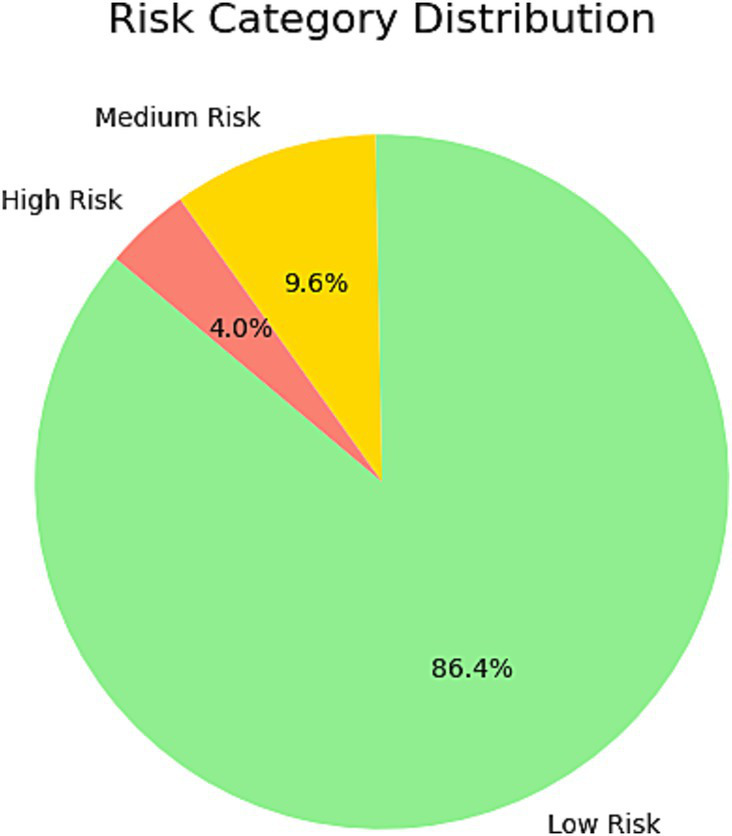
This pie chart illustrates the distribution of risk categories among individuals in the test set as predicted by the model. The chart divides the population into three categories: low risk, medium risk, and high risk. The majority of individuals were classified as Low Risk, reflecting their lower predicted probability of *H. pylori* infection. High-risk individuals accounted for a small proportion, suggesting that these individuals warrant further attention and targeted screening. This classification provides a basis for personalized screening and risk management.

The histogram of predicted probabilities (Figure 6) shows most participants in the low-risk category, while the pie chart (Figure 7) highlights the small but actionable high-risk subgroup warranting focused intervention.

## Discussion

*H. pylori* remains a highly prevalent infection in China (12), affecting nearly half of the population (13). Due to its often silent progression, many cases are diagnosed late, when gastric mucosal damage is already advanced (14, 15). Our findings affirm the systemic impact of chronic infection, linking it to elevated BMI, HbA1c, LDL-C, and inflammatory markers. These associations support the hypothesis that *H. pylori* contributes to metabolic dysregulation through inflammatory and endocrine mechanisms (16–19).

Serum albumin reduction in infected individuals likely reflects a hepatic acute-phase response rather than primary protein-losing conditions (20). In a large retrospective Chinese cohort (*n* = 29,154), serum albumin was inversely associated with acute *H. pylori* infection (*p* < 0.001), supporting its role as an independent marker of systemic inflammation in this setting (16). Elevated WBC and inflammatory albumin derivatives further reinforce this systemic response (21).

Evidence suggests a bidirectional link between *H. pylori* infection and BMI. A meta-analysis of 34 studies (*n* = 175,575) found *H. pylori*–positive individuals had slightly higher BMI than uninfected ones (22). NAFLD patients—often with high BMI—also showed greater infection rates, implying mutual metabolic effects. However, NHANES data (*n* = 1,568) found no link between general obesity and infection, though central adiposity was associated with seropositivity in younger adults (23). These patterns point to a dynamic interplay between infection and metabolic regulation.

Our XGBoost model achieved robust performance, outperforming conventional algorithms in predicting infection risk. SHAP analysis highlighted the role of metabolic variables—particularly HbA1c and albumin—in driving predictions. Model calibration and risk distribution plots demonstrated its utility for stratifying individuals into low-, moderate-, and high-risk categories.

Importantly, AI-based models should complement, not replace, clinical judgment. For high-risk individuals, confirmatory endoscopy remains essential, especially when symptoms or mucosal abnormalities are present (24). Our model aligns with modern risk tools but benefits from its continuous scoring mechanism, unlike static models like the ABC method (25). However, our dataset lacked key behavioral and environmental risk factors—such as smoking, salt intake, and socioeconomic status—which may further improve predictive accuracy in future models.

While our findings are encouraging, limitations include a moderate AUC, cross-sectional design, and single-center cohort. Future work should expand sample size, integrate additional biomarkers, and validate performance across diverse populations.

### Strengths and limitations

A major strength of this study is the 6-year longitudinal UBT dataset, allowing robust identification of chronic infection patterns. The integration of machine learning with traditional biostatistical methods provided both interpretability and predictive accuracy. However, the moderate AUC values suggest that additional predictors, such as microbiome or genetic data, may enhance model performance. Our single-center design may limit generalizability, and external validation in diverse populations is warranted.

## Conclusion

This study underscores the interplay between chronic *H. pylori* infection and metabolic dysfunction, offering new perspectives on risk prediction using machine learning. The XGBoost model demonstrated reliable performance in stratifying infection risk based on accessible clinical markers. Its integration into routine screening protocols could enhance early detection and personalized intervention strategies. Further studies should validate these findings across broader populations and incorporate additional risk factors.

## Data Availability

The raw data supporting the conclusions of this article will be made available by the authors, without undue reservation.
